# In Vitro Effect of *Zingiber officinale* Extract on Growth of *Streptococcus mutans* and *Streptococcus sanguinis*

**DOI:** 10.1155/2015/489842

**Published:** 2015-08-12

**Authors:** Arash Azizi, Shabnam Aghayan, Saeed Zaker, Mahdieh Shakeri, Navid Entezari, Shirin Lawaf

**Affiliations:** ^1^Oral Medicine Department, Islamic Azad University Dental Branch, Tehran, Iran; ^2^Periodontics Department, Islamic Azad University Dental Branch, Tehran, Iran; ^3^Microbiology Department, Islamic Azad University, Parand Branch, Tehran, Iran; ^4^Islamic Azad University Dental Branch, Tehran, Iran; ^5^Prosthodontics Department, Islamic Azad University Dental Branch, Tehran, Iran

## Abstract

*Background and Objectives*. Tooth decay is an infectious disease of microbial origin. Considering the increasing prevalence of antibiotic resistance due to their overuse and also their side effects, medicinal plants are now considered for use against bacterial infections. This study aimed to assess the effects of different concentrations of *Zingiber officinale* extract on proliferation of *Streptococcus mutans* and *Streptococcus sanguinis* in vitro. *Materials and Methods*. In this experimental study, serial dilutions of the extract were prepared in two sets of 10 test tubes for each bacterium (total of 20). Standard amounts of bacterial suspension were added; 100*ƛ* of each tube was cultured on prepared solid agar plates and incubated at 37°C for 24 hours. Serial dilutions of the extract were prepared in another 20 tubes and 100*ƛ* of each tube was added to blood agar culture medium while being prepared. The mixture was transferred to the plates. The bacteria were inoculated on plates and incubated as described. *Results*. The minimum inhibitory concentration (MIC) was 0.02 mg/mL for *S. mutans* and 0.3 mg/mL for *S. sanguinis*. The minimum bactericidal concentration (MBC) was 0.04 mg for *S. mutans* and 0.6 mg for *S. sanguinis*. *Conclusion*. *Zingiber officinale* extract has significant antibacterial activity against *S. mutans* and *S. sanguinis* cariogenic microorganisms.

## 1. Introduction

Tooth decay is an infectious, progressive disease disrupting the normal molecular interactions between the tooth surface and microbial biofilm. If not treated early, tooth decay can result in tooth cavity and subsequent dentin loss and pulp injury [1, 2]. At this point, tooth decay and loss of mineral content (calcium and phosphorous) are irreversible and the lost tooth structure can only be restored with restorative dental materials [3].* Streptococcus mutans* and* Streptococcus sanguinis* belong to the viridans streptococci group, which are the most commonly found members of the normal flora of the oral cavity. These bacteria synthesize large polysaccharides such as dextran and levan from sucrose and play an important role in development of dental caries. Moreover, following an injury or trauma to the mucosa, they may enter the blood stream and cause endocarditis of the heart valves [3, 4].

*Zingiber officinale* plant, commonly known as ginger, has more than 1200 species in 53 genera. It has been used as a medication since ancient times. According to the Chinese Pharmacopoeia, the medicinal uses and indications of ginger include epigastric pain, vomiting, diarrhea, weak pulse, dyspnea, cough, and sputum production [5]. Also, ginger has well recognized antibacterial [6–9], antifungal/antimycotic [10, 11], and anticancer [12] properties.

Considering the previous reports regarding the antimicrobial activities of ethnic medicinal plants in Iran [13] and the increasing prevalence of antibiotic resistance due to their overuse as well as their side effects, researchers have become increasingly interested in medicinal plants to find new sources of antibacterial remedies [14].

Arheumin drop is a formulation of ginger extract produced by Yas Darou Pharmaceuticals (Tehran, Iran) and in case of confirming its antibacterial efficacy can be used as a substitute for chemical antimicrobial agents. This study aimed to assess the antibacterial effect of* Zingiber officinale* extract on* S. mutans* and* S. sanguinis*. The null hypothesis was that* Zingiber officinale* extract would have no antibacterial effect on* S. mutans* and* S. sanguinis*.

## 2. Materials and Methods

This study was approved by the Ethics Committee of Islamic Azad University, School of Dentistry. Standard strains of* S. mutans* (ATCC 35608) and* S. sanguinis* (ATCC 10556) were procured in lyophilized form from the Institute of Standards & Industrial Research of Iran.* Zingiber officinale* Roscoe was also purchased (Arheumin drop, Yas Darou Pharmaceuticals, Tehran, Iran).

In this study, serial dilutions of* Zingiber* extract were prepared in four series of 10 tubes each (20 tubes for* Streptococcus mutans* and 20 tubes for* Streptococcus sanguinis* from 1/2 to 1/1024 for the two assessment methods). In each method of assessment, 20 tubes (10 tubes for the* Streptococcus mutans* and 10 tubes for* Streptococcus sanguinis*) were selected. In all four groups, one tube was considered as the control group. In the control groups in both methods, microorganisms were added to the culture medium with no extract and their growth or inhibition was evaluated.

Eleven test tubes containing 5 mL of saline solution (0.9%) were prepared; extracts at different concentrations were added to 10 test tubes while the 11th tube was considered as the control and inoculated with the microbial suspension with no extract.

The herb extract was passed through a 0.45 *μ* membrane filter for the purpose of sterilization; 5 cc of the extract was added to the first tube to achieve 1/2 dilution (0.6 mg/mL of pure extract). Next,  5 cc of the first tube was transferred to the second tube to achieve 1/4 dilution (0.3 *μ*g/mL of pure extract). Accordingly, 1/2 (0.6 *μ*g of pure extract), 1/4 (0.3 *μ*g of pure extract), 1/8 (0.15 *μ*g of pure extract), 1/16 (0.08 *μ*g of pure extract), 1/32 (0.04 *μ*g of pure extract), 1/64 (0.02 *μ*g of pure extract), 1/128 (0.01 *μ*g of pure extract), 1/256 (0.005 *μ*g of pure extract), 1/512 (0.002 of *μ*g pure extract), and 1/1024 (0.001 *μ*g of pure extract) dilutions (concentrations) of the extract were prepared as such.

We used two different culture methods to eliminate any possible errors.

### 2.1. Method One

*Streptococcus mutans* and* S. sanguinis* were cultured in separate culture media and 0.5 McFarland standard of pure bacteria was added to each tube and mixed. Thus, 10 tubes contained* S. mutans* and 10 tubes contained* S. sanguinis*. Next, 100 *Υ* of the microbial suspension in each tube was cultured in solid blood agar culture medium previously prepared in separate plates. The cultured plates were incubated at 37°C for 24 hours (Memmert, Germany).

### 2.2. Method Two

Serial dilutions of the extract were prepared again in another 20 test tubes and 100 *Υ* of the microbial suspension at each concentration was added to the blood agar culture medium (9.9 cc) while being prepared. Understudy bacteria were cultured in plates containing the solid culture medium and the diluted extract. The plates were incubated at 37°C for 24 hours (Memmert, Germany). Contents of the control tubes were also incubated at the same conditions without inoculation with the microbial suspension.

### 2.3. Determination of MIC

The MIC is defined as the minimum concentration of the extract that completely inhibits visible bacterial growth compared to the negative control group. In our study, the MIC of the* Zingiber officinale* extract was the concentration of the extract in the first plate showing inhibitory effects of the extract on bacterial proliferation.

### 2.4. Determination of MBC

The MBC is defined as the minimum concentration of the extract at which no bacterial growth is observed. In our study, concentration of the extract in the last plate showing no bacterial colony was considered as the MBC of* Zingiber officinale* extract [15].

## 3. Results

After 24 hours, the plates were removed from the incubator and evaluated.

### 3.1. Method One (Simultaneous Culture of Bacterial Suspension and the Extract in Solid Blood Agar Medium)

*Streptococcus mutans* showed no proliferation in plates 1-2 and bacterial colonies gradually appeared in plates 3–10 (Figure 1).

*Streptococcus sanguinis* showed no bacterial proliferation in plate 1 but bacterial colonies appeared in plates 2–10 (Figure 2).

### 3.2. Method Two (Bacterial Culture in the Mixture of Solid Blood Agar Medium and the Diluted Extract)

*Streptococcus mutans* showed no proliferation in plates 1-2 and bacterial colonies gradually appeared in plates 3–10.

*Streptococcus sanguinis* showed no bacterial proliferation in plate 1 but bacterial colonies appeared in plates 2–10.

Control plates in both methods showed proliferation of bacterial colonies.

The MIC of the* Zingiber officinale* extract was 0.02 mg/mL for* S. mutans* and 0.3 mg/mL for* S. sanguinis*.

The MBC of* Zingiber officinale* extract was 0.04 mg/mL for* S. mutans* and 0.6 mg/mL for* S. sanguinis*.

## 4. Discussion

This experimental study sought to assess the antibacterial activity of* Zingiber officinale* extract against* S. mutans* and* S. sanguinis* in vitro. Two methods for bacterial culture were used to eliminate any possible errors. Both methods yielded similar results for both understudy bacteria. In the first method, diluted extracts and the bacterial inoculum were added to the solid blood agar medium and cultured and the obtained MIC and MBC values of the extract were 0.02 and 0.04 mg/mL for* S. mutans* and 0.3 and 0.6 mg/mL for* S. sanguinis*, respectively.

We hypothesized that the solvent used for the experiment can probably influence the final result. In this study, we used ethanol extract of* Zingiber officinale* in order to not corrupt the effective ingredients of the extract.

Kader et al. in 2011 [6] evaluated the antimicrobial effects of ethanolic extract of* Zingiber zerumbet* and its chloroform and petroleum ether soluble fractions against 13 pathogenic bacteria and three fungi using the disc diffusion method. Of the tested solvents of the extract, the ethanol extract had the highest activity against bacteria and fungi. The difference between their study and ours was in the type of understudy microorganisms and the methodology. The disc diffusion method has the advantage of ease of use and concomitant comparison with an antibiotic (kanamycin in their study). However, the advantages of our technique are the elimination of the possibility of procedural errors and uniform distribution of the extract, bacteria, and the culture medium. The reported antibacterial activity and the superiority of the ethanol extract of* Zingiber officinale* were in line with our findings.

The antibacterial effects of* Zingiber officinale* extract observed in our study were in accordance with the results of previous studies. O'Mahony et al., in 2005 [8], evaluated the bactericidal and antiadhesive properties of 25 plants against* Helicobacter pylori*. Their applied method was somehow similar to ours; the only difference was that the amounts of extract, bacteria, and culture medium in their study were different from those in our study. Moreover, the effects were investigated after 60 minutes and no explanation was provided for selection of this specific time period in their study. Type of understudy bacterium (*Helicobacter pylori*) was also different from our understudy bacteria. However, they confirmed the antibacterial activity of* Zingiber officinale*, which is similar to our finding.

In 2012, Gull et al. [7] assessed the inhibitory effects of* Allium sativum* and* Zingiber officinale* extracts on eight drug resistant pathogenic bacteria using the disc diffusion method. The type of understudy bacteria (*Escherichia coli*,* Pseudomonas aeruginosa*,* Bacillus subtilis*,* Staphylococcus aureus*,* Klebsiella pneumoniae*,* Shigella sonnei*,* Staphylococcus epidermidis*, and* Salmonella typhi* in their study) and the methodology of their study were different from those in our investigation. They reported that all the tested bacterial strains in their study were susceptible to both ginger and garlic extracts, which confirms our results.

Karuppiah and Rajaram, in 2012 [16], and Keskin and Toroglu, in 2011 [17], evaluated the antibacterial activity of several plant extracts on some bacterial strains and reported the significant antibacterial effects of* Zingiber officinale* extracts, which is in accordance with our result.

Ewnetu et al., in 2014 [18], assessed the antimicrobial effects of mixtures of Ethiopian honeys and ginger rhizome powder extracts separately and in combination on several pathogenic bacteria. Their results confirmed our findings regarding the favorable antibacterial activity of ginger. Also, they reported the superior efficacy of honey-ginger powder extract mixture due to its therapeutic effects and better taste.

Factors responsible for the high bacterial susceptibility of understudy bacteria to ginger extract have yet to be clearly understood; however, the antibacterial activity of this plant is mainly attributed to its secondary metabolites [19]. Previous studies on the rhizomes of* Zingiber officinale* have revealed that gingerols and shogaols are among the active components of ginger. The characteristic odor and flavor of ginger are caused by a mixture of zingerone, shogaols, and gingerols that are volatile oils which compose 1–3% of the weight of fresh ginger. In animal models, gingerols increased peristalsis and showed analgesic, tranquilizing, antipyretic, and antibacterial properties [20].

The pungent taste of ginger is due to the presence of gingerol with the chemical formula C_17_H_26_O_4_. Ginger contains 3% of fragrant essential oils whose main constituents are sesquiterpenoids with zingiberene being their main component and smaller amounts of other sesquiterpenoids including *β*-sesquiphellandrene, bisabolene, and farnesene and a small monoterpenoid fraction including *β*-phellandrene, cineol, and citral [21].

The spicy and pungent flavor of ginger is due to nonvolatile phenylpropanoid-derived components, especially gingerols and shogaols formed from gingerols when ginger is dried or cooked. By cooking ginger, gingerols are transformed to zingerone, a less pungent compound with a spicy-sweet aroma [22]. The antibacterial effects observed in our study may also be attributed to gingerols. Some studies have demonstrated that the solvent affects the degree of antibacterial activity of extracts and ethanol extract seems to have superior efficacy in this respect. Organic solvents better dissolve organic compounds. Thus, release of active compounds seems to be a necessary requirement for antibacterial activity [23]. In the current study, ethanol extract of ginger was used, which might have also affected the results.

Ginger has no side effects if consumed in rational amounts [24] and is generally recognized as safe by the American Food and Drug Administration. However, it can produce drug interference if consumed with some drugs like warfarin. Its use is also contraindicated in patients with gallstones, because it increases the production of bile [25].

Allergic reactions to ginger may appear as skin rash. Although ginger is recognized to be generally safe, its consumption in large amounts can cause stomach burning, bloating, and nausea, especially when consumed in the form of powder. Not well-chewed fresh ginger may cause intestinal obstruction. Moreover, patients with stomach ulcer, inflammatory intestinal disease, or intestinal obstruction may show allergic reactions to fresh ginger. Also, there are claims that ginger may influence blood pressure and arrhythmia [26].

Considering the reported complications of ginger, in this study we found the exact concentration of* Zingiber officinale* extract that needs to be added to mouth rinses to exert its inhibitory and bactericidal effects on cariogenic bacteria in the oral cavity while preventing the unwanted side effects.

Future studies are required to assess the effect of different fractions of* Zingiber officinale* extract to find its effective component. Moreover, the effect of* Zingiber officinale* extract on other pathogenic bacteria must be investigated. An in vivo study is also required to find the maximum burning effect of ginger on oral mucosa. Last but not least, honey can be added to improve the taste.

## 5. Conclusion

*Zingiber officinale* extract has significant antibacterial effects on* S. mutans* and* S. sanguinis*; however, the latter requires higher concentration of the extract to reach MIC. Considering the obtained MIC and MBC values,* Zingiber officinale* extract can be incorporated into herbal mouth rinses and toothpastes for its antimicrobial effects.

## Figures and Tables

**Figure 1:**
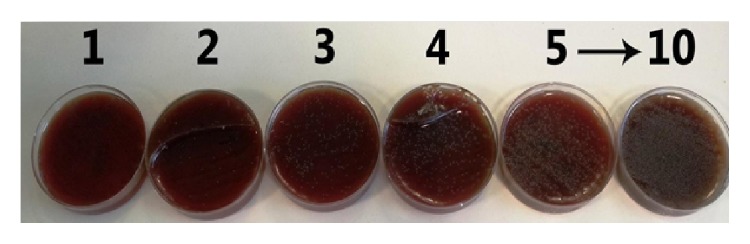


**Figure 2:**
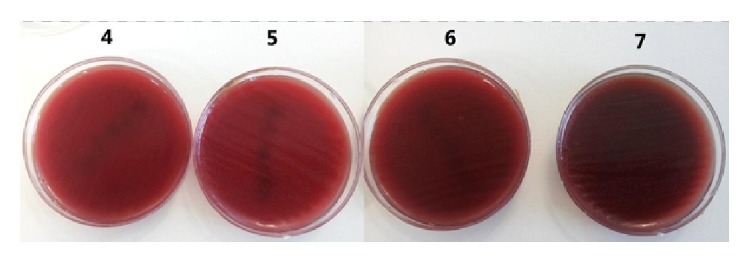
Growth of* Streptococcus sanguis* on plates 4–7.

## References

[1] García-Cortés J. O., Medina-Solís C. E., Loyola-Rodriguez J. P. (2009). Dental caries' experience, prevalence and severity in Mexican adolescents and young adults. *Revista de Salud Pública*.

[2] Jain M., Mathur A., Sawla L. (2009). Oral health status of mentally disabled subjects in India. *Journal of Oral Science*.

[3] Acevedo A. M., Montero M., Machado C., Sáez I., Rojas-Sánchez F., Kleinberg I. (2013). Dental caries experience in school children and the impact of non-cavitated lesions on the caries index. *Acta Odontológica Latinoamericana*.

[4] Nomura R., Naka S., Nemoto H. (2013). Potential involvement of collagen-binding proteins of *Streptococcus mutans* in infective endocarditis. *Oral Diseases*.

[5] Torkzadeh-Mahani S., Nasri S., Esmaeili-Mahani S. (2014). Ginger (zingiber officinale roscoe) prevents morphine-induced addictive behaviors in conditioned place preference test in rats. *Addiction & Health*.

[6] Kader G., Nikkon F., Rashid M. A., Yeasmin T. (2011). Antimicrobial activities of the rhizome extract of Zingiber zerumbet Linn. *Asian Pacific Journal of Tropical Biomedicine*.

[7] Gull I., Saeed M., Shaukat H., Aslam S. M., Samra Z. Q., Athar A. M. (2012). Inhibitory effect of Allium sativum and *Zingiber officinale* extracts on clinically important drug resistant pathogenic bacteria. *Annals of Clinical Microbiology and Antimicrobials*.

[8] O'Mahony R., Al-Khtheeri H., Weerasekera D. (2005). Bactericidal and anti-adhesive properties of culinary and medicinal plants against *Helicobacter pylori*. *World Journal of Gastroenterology*.

[9] Nostro A., Cellini L., Di Bartolomeo S. (2006). Effects of combining extracts (from propolis or *Zingiber officinale*) with clarithromycin on *Helicobacter pylori*. *Phytotherapy Research*.

[10] Ali B. H., Blunden G., Tanira M. O., Nemmar A. (2008). Some phytochemical, pharmacological and toxicological properties of ginger (*Zingiber officinale* Roscoe): a review of recent research. *Food and Chemical Toxicology*.

[11] Giriraju A., Yunus G. Y. (2013). Assessment of antimicrobial potential of 10% ginger extract against *Streptococcus mutans*, *Candida albicans*, and *Enterococcus faecalis*: an in vitro study. *Indian Journal of Dental Research*.

[12] Park Y. J., Wen J., Bang S., Park S. W., Song S. Y. (2006). [6]-Gingerol induces cell cycle arrest and cell death of mutant p53-expressing pancreatic cancer cells. *Yonsei Medical Journal*.

[13] Pirbalouti A. G., Siahpoosh A., Setayesh M., Craker L. (2014). Antioxidant activity, total phenolic and flavonoid contents of some medicinal and aromatic plants used as herbal teas and condiments in Iran. *Journal of Medicinal Food*.

[14] Nariman F., Eftekhar F., Habibi Z., Falsafi T. (2004). Anti-*Helicobacter pylori* activities of six Iranian plants. *Helicobacter*.

[15] Bali E. B., Açik L., Akca G. (2014). Antimicrobial activity against periodontopathogenic bacteria, antioxidant and cytotoxic effects of various extracts from endemic *Thermopsis turcica*. *Asian Pacific Journal of Tropical Biomedicine*.

[16] Karuppiah P., Rajaram S. (2012). Antibacterial effect of Allium sativum cloves and *Zingiber officinale* rhizomes against multiple-drug resistant clinical pathogens. *Asian Pacific Journal of Tropical Biomedicine*.

[17] Keskin D., Toroglu S. (2011). Studies on antimicrobial activities of solvent extracts of different spices. *Journal of Environmental Biology*.

[18] Ewnetu Y., Lemma W., Birhane N. (2014). Synergetic antimicrobial effects of mixtures of Ethiopian honeys and ginger powder extracts on standard and resistant clinical bacteria isolates. *Evidence-Based Complementary and Alternative Medicine*.

[19] Nweze E. I., Okafor J. I., Njoku O. (2004). Antimicrobial activities of methanolic extracts of *Trema guineensis* (Schumm and Thorn) and *Morinda lucida* Benth used in Nigerian. *Journal of Biological Research and Biotechnology*.

[20] O'Hara M., Kiefer D., Farrell K., Kemper K. (1998). A review of 12 commonly used medicinal herbs. *Archives of Family Medicine*.

[21] Bisset N. G. (1994). *Herbal Drugs and Phytopharmaceuticals: A Handbook for Practice on a Scientific Basis*.

[22] Wood G. (2013). Class IX. Sialagogues. *A Treatise on Therapeutics, and Pharmacology or Materia Medica, Volume 2*.

[23] Ekwenye U. N., Elegalam N. N. (2005). Antibacterial activity of ginger (*Zingiber officinale* Roscoe) and garlic (*Allium sativum* L.) extracts on *Escherichia coli* and *Salmonella typhi*. *International Journal of Molecular Medicine and Advance Sciences*.

[24] Marcello S. (2001). *The Psychopharmacology of Herbal Medications: Plant Drugs That Alter Mind, Brain, and Behavior*.

[25] Al-Achi A. (2007). *A Current Look at Ginger Use*.

[26] Mayo C. (2007). *Drugs & Supplements: Ginger (Zingiber Officinale Roscoe)*.

